# A Preliminary Study of the Occurrence of Genetic Changes in mtDNA in the Muscles in Children Treated for Strabismus

**DOI:** 10.3390/jcm13144041

**Published:** 2024-07-10

**Authors:** Wojciech Pawłowski, Joanna Reszeć-Giełażyn, Marzanna Cechowska-Pasko, Beata Urban, Alina Bakunowicz-Łazarczyk

**Affiliations:** 1 Department of Pediatric Ophthalmology and Strabismus, Medical University of Białystok, 15-089 Białystok, Poland; wojciech.pawlowski@umb.edu.pl (W.P.); beata.urban@umb.edu.pl (B.U.); 2 Department of Medical Pathomorphology, Medical University of Białystok, 15-089 Białystok, Poland; joanna.reszec-gielazyn@umb.edu.pl; 3 Department of Pharmaceutical Biochemistry, Medical University of Białystok, 15-089 Białystok, Poland

**Keywords:** mtDNA, extraocular muscles, strabismus, ANT1

## Abstract

**Background:** The dysregulation of extraocular muscles (EOMs) in the strabismus may be partly due to modification in the mitochondrial DNA (mtDNA). Currently, little is known about changes occurring in mtDNA of EOMs in patients with strabismus, therefore the aim of our study was to analyze if there are any changes occurring in the mitochondrial DNA of extraocular muscles in children that underwent strabismus surgery in our clinic. **Methods:** MtDNA was isolated from the tissue material using the Qiagen kit. Assessment of mtDNA mutations was performed by next-generation sequencing (NGS) using the Illumina MiSeq protocol. **Results:** The examination revealed the presence of atrophic changes in muscle fibers. NGS evaluation revealed a dominant genetic mutation in the ANT1 gene in 12 of the 15 patients examined. **Conclusions:** The presented results constitute the beginning of research on changes in mtDNA occurring in the muscles of children with strabismus surgery. Further studies are necessary in the context of resolving the transcriptomic differences between strabismic and non-strabismic EOMs. Better understanding of the molecular genetics of strabismus will lead to improved knowledge of the disease mechanisms and ultimately to a more effective treatment.

## 1. Introduction

Changes in the extraocular muscles (EOM) in patients with strabismus are still being investigated. Better understanding of muscle cell biology in patients with strabismus may suggest alternative approaches to the treatment of such disorders.

Strabismus is a condition in which the eyes are not properly aligned with each other. It typically involves a lack of coordination between EOMs. Misalignment between the eyes can be caused by a dysfunction of at least one of the six muscles responsible for the movement of the eyes. The six extraocular muscles in each eye have to work symmetrically and with forces which are equally applicated to cause their movement. If there is an imbalance in application of those forces to EOMs, a misalignment between the eyes occurs.

Strabismus surgery involves the extraocular muscles and is performed to correct eye misalignment. The procedure is performed under general anesthesia. Various factors may cause eye misalignment. Surgical treatment of the strabismus is indicated when other non-invasive methods are unable to treat the misalignment. The type of surgery depends on the type of strabismus. Esotropia is a misalignment in which one eye is deviated inward (convergent strabismus). Exotropia is a type of eye misalignment, where one eye deviates outward (divergent strabismus). Correction of strabismus can be achieved by loosening or tightening the extraocular muscle in order to weaken or strengthen them, respectively [1]. After the specific ophthalmological examination and orthoptic measurements, the appropriate amount of weakening or strengthening is determined. The procedure performed to weaken a rectus muscle is called recession, where a muscle is cut off from the eye and sewn further back onto the eyeball. Resection and plication are procedures which are used to strengthen the rectus muscles. In resection, a small fragment of the muscle is cut away and then it is reattached to its original position. In the procedure of plication, the muscle is folded and secured to the sclera.

ADP/ATP translocase 1 or adenine nucleotide translocator 1 (ANT1) is an enzyme that in humans is encoded by the SLC25A4 gene. ANT1 forms a channel in the inner mitochondrial membrane. This channel allows ADP to pass into the mitochondria and ATP from the mitochondria which is ready to be used as energy for the cell. ANT1 can also be part of another structure in the inner membrane called the mitochondrial permeability transition pore. This structure allows various molecules to pass into the mitochondria and is thought to play a role in cell apoptosis. Mutations which occur in the gene responsible for encoding ANT1 have been linked to degenerative conditions that can affect the muscles and the brain. However, it remains unclear how these mutations cause the disease. The findings of Coyne et al. suggest that certain genetic mutations in the gene that encodes the ANT1 protein cause the disease by preventing and blockading the transport of other proteins into mitochondria, rather than directly influencing ANT1’s nucleotide transport role in the cell [2]. It has a great impact on the understanding of diseases associated with mitochondrial proteins. It can potentially alter the way in which the treatments for these conditions are designed. There are references that several missense mutations in ANT1 caused dominant pathology. These ANT1 mutations cause adult- and late-onset disease, autosomal dominant Progressive External Ophthalmoplegia (adPEO), which is manifested by ptosis, ophthalmoplegia, and skeletal muscle weakness together with multiple mitochondrial DNA (mtDNA) deletions. Patients suffering from dominant ANT1 mutations can also present neurological symptoms such as sensorineural hearing loss, clonic seizures, bipolar disorder, cortical atrophy, and dementia [3].

Currently, little is known about the changes that occur in the mtDNA of the extraocular muscles in patients with strabismus, therefore the aim of our study was to analyze the changes that occur in the mtDNA in the extraocular muscles in children operated due to strabismus in our clinic. We hypothesized that the dysregulation of EOMs in the strabismus may be partly due to modification in mtDNA, so the objective of this study was to determine whether mutations in mtDNA can be observed in EOMs of children with strabismus. The inclusion criteria were accommodative strabismus and infantile strabismus. The exclusion criteria were strabismus that occurred with associated syndromes, e.g., Moebius syndrome, strabismus that occurred in children born prematurely, and strabismus in children with additional neurological diseases.

## 2. Materials and Methods

### 2.1. Study Subjects

This study was conducted using postoperative material from the Department of Pediatric Ophtalmology and Strabismus of the Medical University of Bialystok in Poland. The materials from the patients were collected from 15 children operated on due to strabismus in our clinic. Extraocular muscles during the procedure were secured in liquid nitrogen and then stored in a −80 °C freezer. Some of the material was secured for histopathological examination to assess the morphology of muscle fibers.

### 2.2. Preparation of Enriched mtDNA Samples

MtDNA was isolated from tissue material using the Qiagen kit. The crushed tissue from the collected muscle sections (approx. 50 mg) was homogenized in homogenization buffer (i.e., 0.25 sucrose, 10 mM EDTA, 30 mM Tris/HCl, pH = 7.5). The homogenates were transferred to 1.5 mL tubes, centrifuged at 1000× *g* at 4 °C for 1 min to deposit nuclei and cell fragments. The resulting supernatant was centrifuged (12,000× *g*, 4 °C, 10 min) to settle the mitochondria. Obtained after centrifugation, the supernatants were removed and the pellets containing mitochondria were suspended in buffer (i.e., 0.15 M NaCl, 10 mM EDTA, 10 mM Tris/HCl, pH = 8.0). The total volume of the solution was 50 µL. Then, 100 µL of NaOH in SDS was added (i.e., freshly prepared 0.18 M NaOH solution in 1% SDS solution). The samples were shaken briefly on a Vortex shaker. After shaking, samples were incubated on ice for 5 min, and then 75 μL of alkaline lysis solution (i.e., 3M potassium acetate solution vs. potassium and 5M relative to acetate: add 11.5 mL of ice-cold CH_3_OOH to 60 mL of 5M CH_3_COOK solution and 28.5 mL of H_2_O) cooled to approximately 0 °C. The samples were again gently shaken with the Vortex device and incubated on ice (5 min). After incubation, the samples were centrifuged at 12,000× *g* at 4 °C for 5 min. The resulting supernatants were collected. An equal volume of phenol/chloroform/isoamyl alcohol mixture was added to the supernatants (25:24:1). The mixture was mixed vigorously by shaking with the Vortex and then it was centrifuged (12,000× *g*, RT, 2 min). The obtained aqueous phase was transferred to a new test tube with 2 volumes of cooled absolute ethanol, mixed by shaking and allowed to stand at RT for approx. 15 min. After incubation, the sample was centrifuged (12,000× *g*, RT, 2 min). The obtained mtDNA pellet was washed in 1 mL of 70% ethanol solution. The precipitate was dried for 1–3 min under reduced pressure. The dried pellet was dissolved in TE buffer containing RNase (i.e., RNase solution with a concentration of 20 µg/mL in TE buffer at pH = 8: 10 mM Tris’HCl, 1 mM).

### 2.3. NGS Sequencing of mtDNA

The amount of deleted mtDNA and mtDNA copy numbers were obtained by real-time quantitative PCR. Quantification of cDNAs for ANT1, TYMSF, and PEO1 were performed by real-time quantitative PCR.

ANT1, TYMS, and PEO1 genes were performed by PCR according to the protocol: sterile ultra-pure water; dNTP mix, Taq DNA polymerase, 10× PCR assay buffer, MgCl_2_ (50 mM); primers for ANT1, TYMS, and PEO1. The reaction was subjected to an initial denaturation step (94.0 °C for 5 min), followed by 35 cycles of denaturation (94.0 °C for 1 min), primer annealing (59.1 °C for 1 min), and extension (72.0 °C for 2 min), and 10 min of final incubation at 72.0 °C. Analysis of the PCR product was performed by electrophoresis on a 12% polyacrylamide gel. 

The mtDNA obtained this way was subjected to sequence analysis using Illumina NGS kits. The mtDNA was sequenced using the TruSeq PCR free library preparation kit (Illumina, San Diego, CA, USA), and sequencing used the Illumina HiSeq X platform (Illumina, San Diego, CA, USA) to identify deletions and duplications in the ANT1, TYMS, and PEO1 gene. An NGS panel consisting of 281 nuclear genes encoding mitochondrial proteins involved in the most common mitochondrial pathologies was used to screen for mutations. 

Library preparation for each sample was carried out using a TruSeq PCR free library preparation kit (Illumina, San Diego, CA, USA). All identified variants were verified by Sanger sequencing. After PCR amplification, each PCR product was purified and then analyzed by direct sequencing. Analysis of variants, including non-synonymous variants, splice sites variants, and small indels, was compared with the dbSNP data baseband in the Human Gene Mutation Database, as well as by a literature review. Variants of interest were confirmed in probands and then in their parents or siblings using Sanger sequencing.

## 3. Results

**Figure 1:**
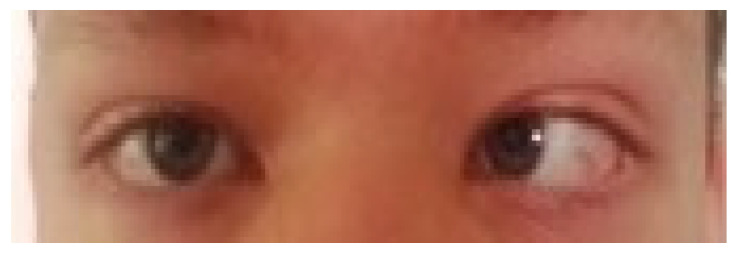
Child before convergent strabismus surgery.

**Figure 2:**
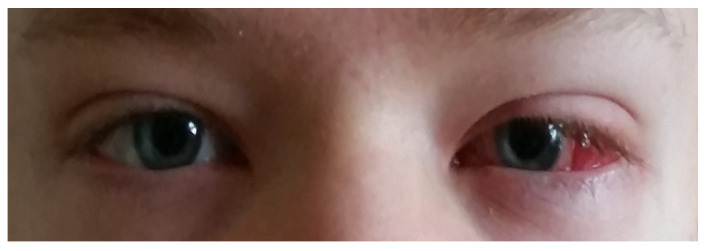
Child after convergent strabismus surgery.

The extraocular muscles were collected from 15 patients; 8 of them were operated on for convergent strabismus and 7 patients were operated on for divergent strabismus. Data of the operated children are presented in Table 1 and Table 2. Medial rectus muscles were resected and then examined in patients with divergent strabismus and lateral rectus muscles were resected and then examined in patients with convergent strabismus. In patients with large strabismus angle deviation apart from resection of the part of the extraocular muscle, recession of the opposite muscle in the same eye was performed (Figure 1 and Figure 2).

Histopathological evaluation of EOMs showed mostly normal muscle fibers. Only 3 out of 15 lesions have shown focal signs of atrophic muscle fibers. In two patients operated on for convergent strabismus, we observed the features of atrophy of muscle fibers in the marginal part of the bundle (Table 1), and in one case, we have shown the features of focal atrophy of muscle fibers in the marginal part that occurred in patients operated on due to divergent strabismus (Table 2).

No mutations were found in the TYMP and PEO1 genes. The presence of point mutations in the ANT1 gene was found in 12 of 15 examined patients (deletions or base substitutions). In our study, we observed G deletion (three cases), C deletion (one case), A deletion (one case), and in one case, T deletion and C>A base substitution in patients operated on for convergent strabismus. In two cases, we did not observe any signs of mutation (Table 1). Mutations in mtDNA in patients operated on for divergent strabismus have shown a T deletion in three cases, a C>A base substitution in two cases, and a G deletion and a C>A base substitution in one case. In one case, we observed no signs of mutation (Table 2).

## 4. Discussion

### 4.1. Anatomy and Physiology of Extraocular Muscles

Skeletal muscles generally perform specific limited roles whereas EOMs have to be responsive over a wider dynamic range. As a result structural, functional, biochemical, and immunological properties of EOMs are fundamentally different compared to other skeletal muscles [4] EOMs show some interesting anatomical and physiological differences compared to other skeletal muscles. Extraocular muscle fibers are smaller and contract more than 10 times faster than other skeletal muscles. These muscle fibers are highly innervated. One nerve fiber innervates seven muscle fibers (1:7), whereas in other skeletal muscles, one nerve fiber innervates up to a 100 muscle fibers [5]. Another distinction is the presence of two distinct muscle types: the fast or twitch fibers that are innervated by a single large motor neuron with “en paque” neuromuscular junctions, and the slow or tonic fibers that are innervated by multiple small-diameter motor nerves with “en grappe” neuromuscular endings. Another difference between EOMs and other muscles is the large amount of elastic tissue found interspersed in elastic bands throughout the muscle. Janbaz et al. studied the distribution of the intermediate filament proteins desmin, vimentin, and nestin in human EOMs and they have noticed that they differed significantly from the other body muscles with respect to their intermediate filament composition [6].

### 4.2. Molecular Specificity of Extraocular Muscles

In addition to functional differences between extraocular and skeletal muscles, there are also molecular differences such as a higher proportion of fast myosin heavy chain isoforms, specific myosin heavy chain isoforms: extraocular (MyHC-eom) and tonic myosin heavy chain isoforms (MyHC-sto), continuous expression of developmental isoforms (MyHC-emb and MyHC-pn), and higher density of sarcoplasmic reticulum ion pumps (SERCA) in extraocular muscle fibers [7].

### 4.3. Extraocular Muscles of Adults with Strabismus

Little is known about the changes in the EOMs in patients suffering from strabismus. Unfortunately, the etiology of most forms of strabismus is unknown, a better understanding of muscle cell biology in patients with strabismus can lead to alternative approaches to treating these conditions. It was found that in muscles of patients with strabismus, who had underacting medial rectus muscles, the total number of activated and satellite cells was upregulated based on the number of myofibers in cross-sections, compared with the number in normal medial rectus muscles from age-matched control eyes [8]. Change in the antioxidative capacity of extraocular muscles in patients with exotropia is one example of the described alteration. Medial rectus muscles of patients with constant exotropia had a redox imbalance status: the neuronal nitric oxide synthase level was significantly higher in the exotropic group than in the control group [9]. On the other hand, a catalase expression level was higher in the control group than in the exotropic group. Li et al. evaluated the morphological changes of extraocular muscle proprioceptors in concomitant strabismus [10]. Under electron microscopy, they observed that the general architecture of the receptors in concomitant strabismus was completely disorganized and the nerve component in them had disappeared. Additionally, the number of the mitochondria of the axon in the experimental group was significantly lower than that in the control group. In their opinion, these results show that in concomitant strabismus both reception and transmission of proprioceptive information are abnormal, and also the morphological extraocular muscle proprioceptor disturbance plays an important role in the pathogenesis of concomitant strabismus.

The extracellular matrix is important for elasticity and the tension of the tissue and it may play some role in the development of strabismus. Yamane et al. measured the amounts of aggrecan, fibronectin, and laminin in the medial rectus muscle of adult patients with intermittent exotropia to understand the importance of extracellular matrix components [11]. The total amount of aggrecan, fibronectin, and laminin in the excised tissue was correlated with the clinical data of the patients such as age, exodeviation, and refractive error. The amount of aggrecan significantly decreased with age, while the amount of laminin or fibronectin did not correlate with age. Patients with a basic type of intermittent exotropia showed a tendency to develop larger amounts of aggrecan than patinets with a convergence insufficiency type. Those differences were not statistically significant. They concluded that the amount of aggrecan can be related to motor aspects of intermittent exotropia. Comparable observation about the role of the extracellular matrix molecules in the intermittent and constant exotropia was made by Liu et al. [12]. They measured the amounts of fibronectin and proteoglycan in the resected medial rectus muscles of patients with concomitant exotropia. The amount of fibronectin in the resected medial rectus muscle of patients with concomitant exotropia was significantly lower than that observed in normal individuals. Patients with intermittent exotropia showed a significantly higher concentration of fibronectin than those with constant exotropia and the amount of proteoglycans had no significant difference between the two groups. In contrast, Zuo et al. studied resected medial rectus muscles in patients with concomitant exotropia and the amounts of fibronectin in the resected medial rectus muscle of patients with constant exotropia were significantly lower than those of the control and intermittent exotropic groups, while patients with intermittent exotropia did not show significantly lower amounts of fibronectin than those observed in the control group [13].

### 4.4. Extraocular Muscles in Pediatric Strabismus

Quantitative findings indicate that there are minimal but persistent morphological changes exist between strabismic and non-strabismic EOMs of children with strabismus. The significance of these changes in the pathogenesis of strabismus remain unclear. Light microscopy of 90 biopsy specimens from 80 children with strabismus showed significant variation in muscle fiber shape and size with disruption of sarcomeres, a prominent increase in endomysial and perimysial collagen, numerous vacuoles, and subsarcolemma inclusions. Electron microscopy examination showed disruption of myofilaments, nemaline rods, abnormal mitochondria, leptomer profiles, occasional “myelin figures”, glycogen, and lipid-like droplets. A few intramuscular nerves contained long-spacing collagen (“Luse bodies”). In contrast to somatic skeletal muscle, observation based on enzymatic histochemistry revealed that EOMs showed a consistent lack of mosaic pattern and reciprocal stain activity between fiber types [14].

The ultrastructure of the extraocular muscles in patients with congenital strabismus is not fully understood and the structures responsible for the pathogenesis of this condition are still to be determined. One of them can be innervated myotendinous cylinders (IMCs), found in the distal myotendinous regions of EOMs. The IMCs of patients with acquired strabismus did not show significant morphological changes. However, significant IMC changes were observed at the distal myotendinous junction of patients with congenital strabismus [15]. Domenici-Lombardo L et al. suggested that the most important functional alteration in strabismus regards the scleral myotendinous junction [16]. Another alteration observed in congenital strabismus is concerned with extraocular muscle proprioceptor impairment. The sensorial structures which are located at the myotendinous junction of the EOMs of patients suffering from congenital strabismus had significant alterations: the receptors are smaller, with an inner capsule dividing the tendinous component into irregularly shaped compartments and changes in their general architecture [17]. Kim et al. performed ultrastructural examination of extraocular muscle tendon axonal profiles in infantile and intermittent exotropia [18]. They noted many axonal degenerative findings, such as the retraction of axons from myelin sheaths with considerable shrinkage, axonal disintegration, and Schwann cell proliferation in muscles of patients with infantile exotropia. They also identified three unique findings in muscles of patients with intermittent exotropia: intact axons with incomplete Schwann cell wrapping, intact Schwann cells not associated with axons, and disorganized Schwann cells with shrunken axons. Moreover, Schwann cell degeneration of tendon proprioceptors in the medial rectus might induce the degeneration of proprioceptors in patients with intermittent exotropia over time [19].

For most cases of childhood strabismus, the reason for the imbalance between EOMs is unclear. Strabismus may have a genetic component and susceptibility loci for strabismus have been reported based on analysis of families with inherited forms of strabismus [20]. Non-syndromic infantile esotropia may be associated with chromosomal regions 3p26.3–26.2 and 6q24.2–25.1 and may share alleles that underlie Duane retraction syndrome [21].

### 4.5. Molecular Genetics of Strabismus

It has been suggested that the differences in gene expression may provide information about the causes and consequences of the strabismus condition in humans [22]. Until 2005, 338 genes were found to be differentially expressed in EOMs [22]. The gene expression profile of these two types of rectus EOMs (strabismic vs. normal) has been examined recently. Altick et al. were the first; in 2012, they examined EOM samples obtained during corrective surgery from patients with horizontal strabismus and from deceased organ donors with normal extraocular muscles [23]. Microarray analysis revealed that 604 genes had significantly different expression in these samples. The expression was predominantly upregulated in genes involved in an extracellular matrix structure and downregulated in genes involved in incontractility. Expression of genes involved in signaling, calcium handling, mitochondrial function and biogenesis, and energy homeostasis also differed significantly between normal and strabismic EOMs. The observed decrease in the expression of contractility genes and increase in the number of extracellular matrix-associated genes indicate an imbalance in the structure of EOMs. Real-time qPCR analysis has shown an upregulation of the expression of insulin-like growth factor 1 (*IGF1*)—which regulates mass and contraction kinetics of EOMs. The authors of the study concluded that gene regulation of proteins important for contractile mechanics and extracellular matrix structure is involved in the pathogenesis and/or consequences of strabismus, suggesting possible new therapeutic targets. On the other hand, Zhu et al. evaluated the expression of seven myogenesis-related genes involved in EOM development (myogenic differentiation 1—MYOD1, myogenin—MYOG, retinoblastoma 1—RB1, cyclin-dependent kinase inhibitor 1A—P21, cyclin-dependent kinase inhibitor 1C—P57, IGF1 and muscle creatine kinase—MCK). In this respect, real-time qPCR analysis of 18 resected EOMs of patients with concomitant strabismus and 12 normal control muscle samples has been conducted [24]. The expression levels of these seven genes in the 18 strabismic EOMs were abnormal. The expression levels of all the genes, with the exception of P57, were reduced in most of the strabismic muscle tissues. These results indicate that the abnormal expression of these myogenesis-related genes can be related to concomitant strabismus. There are also other differences in gene and protein expression between human lateral and medial rectus muscle tissue excised from strabismic patients. Resected tissue from the lateral and medial rectus muscles had different levels of expression of tissue inhibitors of metalloproteinases (TIMP-1 and 2), matrix metalloproteinase (MMP-2), and bone morphogenetic protein (BMP-4). These molecular differences may underlie different characteristics of the two EOMs [25].

The mitochondrial adenine nucleotide transporter type 1 (ANT1) provides an example of a particularly rare class of nuclear genes: one that can cause disease via destabilization of the mtDNA [26]. Specific mutations in the gene encoding ANT1 have been connected to degenerative conditions that influence the muscles and the brain [2]. A role for multi-functional protein ANT1 in human pathogenesis was subsequently confirmed when studies of Italian families with a form of inherited progressive external ophthalmoplegia (PEO) indicated that mutations in ANT1 gene were linked with this disorder. Arbogast et al. hypothesized that ANT1 may contribute to mitochondrial dysfunction and oxidative stress in facioscapulohumeral muscular dystrophy (FSHD) muscle cells by modifying their bioenergetic profile [27]. FSHD is the third most common inherited neuromuscular disorder, which is characterized by progressive weakness and atrophy of facial and shoulder girdle muscles which can also spread to the lower extremity muscles.

In the current study, the presence of point mutations in the ANT1 gene was found in 12 of 15 examined patients. To our knowledge, this is the first research on the subject of mtDNA in strabismic muscles. The presented results are the beginning of research on changes in mtDNA which occurs in muscles of children operated on due to strabismus. The research is still ongoing, and the NGS study will assess changes in mtDNA exons.

Further studies are necessary in the context of resolving the transcriptomic differences between strabismic and non-strabismic EOMs. A better understanding of the molecular genetics of strabismus will lead to improved knowledge of the mechanisms of this disease and ultimately provide a more effective treatment.

## Figures and Tables

**Table 1:** Assessment of changes that occurred in patients operated on due to convergent strabismus; angle of deviation above 10 degrees. (OS—left eye; OD—right eye; ANT1—ADP/ATP translocase 1; C—cytosine; G—guanine; A—adenine; T—thymine).

Angle of Deviation above 10 Degrees					
Gender	DOB	Date of Surgery	Type of Surgery	Morphological Assessment	mtDNA Mutation Assessment
M	October 2002	15 July 2014	Strabismus convergens OS: medial rectus muscle recession, lateral rectus muscle resection	Without pathological changes	ANT1 del C
M	February 2008	9 June 2014	Strabismus convergens OS: medial rectus muscle recession, lateral rectus muscle resection	Without pathological changes	No signs of mutation
M	July 2010	3 June 2014	Strabismus convergens OS: medial rectus muscle recession, lateral rectus muscle resection	Without pathological changes	No signs of mutation
M	December 2005	20 May 2014	Strabismus convergens OD: medial rectus muscle recession, lateral rectus muscle resection	Without pathological changes	ANT1 del G
F	November 2011	4 June 2014	Strabismus convergens OD: medial rectus muscle recession, lateral rectus muscle resection	Features of low-grade muscle fiber atrophy	ANT1 del A
F	July 2009	21 May 2014	Strabismus convergens OS: medial rectus muscle recession, lateral rectus muscle resection	Features of atrophy of muscle fibers in the marginal part of the bundle	ANT1 del G
F	July 2003	26 August 2014	Strabismus convergens OS: medial rectus muscle recession, lateral rectus muscle resection	Without pathological changes	ANT1 del T, ANT1 C>A
F	November 2008	12 August 2014	Strabismus convergens OS: medial rectus muscle recession, lateral rectus muscle resection	Without pathological changes	ANT1 del G

2 prism dioptre ≈ 1 degree deviation.

**Table 2:** Assessment of changes that occurred in patients operated on due to divergent strabismus; angle of deviation below 10 degrees. (OS—left eye; OD—right eye; ANT1—ADP/ATP translocase 1; T—thymine; C—cytosine; A—adenine; G—guanine).

Angle Deviation below 10 Degrees					
Gender	DOB	Date of Surgery	Type of Surgery	Morphological Assessment	mtDNA Mutation Assessment
F	October 2002	22 July 2014	Strabismus divergens OD: medial rectus muscle resection	Features of focal atrophy of muscle fibers in the marginal part	ANT1 del T
M	September 2009	28 May 2014	Strabismus divergens OD: medial rectus muscle resection	Without pathological changes	ANT 1 C>T bp 269
M	December 2007	13 August 2014	Strabismus divergens OS: medial rectus muscle resection	Without pathological changes	ANT1 del T, ANT1 C>A
F	October 2010	23 July 2014	Strabismus divergens OS: medial rectus muscle resection	Without pathological changes	ANT1 del G
F	August 2011	8 July 2014	Strabismus divergens OD: medial rectus muscle resection	Without pathological changes	ANT 1 C>T bp 269
F	November 1999	11 June 2014	Strabismus divergens OS: medial rectus muscle resection	Without pathological changes	ANT1 del T
M	February 2002	12 August 2014	Strabismus divergens OS: medial rectus muscle resection	Without pathological changes	No signs of mutation

2 prism dioptre ≈ 1 degree deviation.

## Data Availability

Data are contained within the article.
